# The Impact of Occupational Noise on Hypertension Risk: A Case-Control Study in Automobile Factory Personnel

**DOI:** 10.3389/fcvm.2022.803695

**Published:** 2022-02-17

**Authors:** Xiaomei Wu, Chaoxiu Li, Xiaohong Zhang, Yumeng Song, Dan Zhao, YueYan Lan, Bo Zhou

**Affiliations:** ^1^Department of Clinical Epidemiology and Center of Evidence Based Medicine, The First Hospital of China Medical University, Shenyang, China; ^2^Department of Clinical Epidemiology, The Fourth Affiliated Hospital of China Medical University, Shenyang, China

**Keywords:** hypertension, noise, dose effect relationship, epidemiology, risk factors

## Abstract

**Background:**

Many epidemiological studies have investigated the relationship between occupational noise and hypertension, but with conflicting findings. This study aimed to assess the relationship between occupational noise exposure and the risk of hypertension.

**Methods:**

A case-control study was conducted to explore hypertension predictors, and then sensitivity analysis was performed based on propensity score matching (PSM). Data were collected from participants' annual physical examinations and occupational noise exposure measurements. Odds ratios (ORs) and 95% confidence intervals (CIs) were estimated using logistic regression analysis. A restricted cubic spline (RCS) function was used to fit the dose-effect relationship.

**Results:**

500 cases and 4,356 controls were included in the study. Multivariate logistic regression showed that an increase in the level of occupational noise [range 68–102 dB(A)] of 1 dB(A), corresponded to an increase in hypertension risk of 8.3% (OR: 1.083, 95% CI: 1.058–1.109). Compared to the first quartile, the risk of hypertension in the fourth quartile was 1.742 (95% CI: 1.313–2.310). After applying PSM to minimize bias, we obtained a population of 500 cases and 1,000 controls. Noise level was significantly associated with the risk of hypertension. In addition, the RCS curve showed the risk of hypertension was relatively stable until a predicted noise level of around 80 dB(A) and then started to increase rapidly afterward (*P*_nonlinear_ = 0.002).

**Conclusions:**

Occupational noise exposure was significantly associated with hypertension risk and there was a positively correlated dose-response relationship.

## Introduction

Noise exposure is a common occupational hazard. Noise exposure not only adversely affects health, but also increases the risk of diseases such as hearing loss (1), ischemic heart disease (2), mental distress, and sleep disturbance (3). To protect workers, an occupational exposure limit has been set for the workplace (standard GBZ2.2-2007) of 85 dB(A) (4). However, even if occupational noise exposure does not exceed this limit, increased incidences of chronic diseases such as hypertension have been shown among workers as revealed by annual physical examinations (5). Hypertension is a serious medical condition associated with increased disease risk to the heart, brain, kidneys, and other organ systems. The burden of hypertension disproportionately affects workers in low and middle-income countries, with two-thirds of cases being found there, largely due to recent increases in risk factors in these populations (6). Previous studies have focused on the typical risk factors for hypertension, such as salt intake (7), smoking (8), and obesity (9), with some studies also addressing the impact of noise exposure on the incidence and development of hypertension (10–15).

Evidence from animal and human studies has suggested hyperactivity of the sympathetic nervous system to be a possible mechanism for the association between noise exposure and hypertension (16). The sympathetic nervous system plays an important role in long-term blood pressure regulation in both normotension and hypertension (17). Noise exposure increases sympathetic activity triggering the release of stress hormones, such as epinephrine and norepinephrine, which act on the corresponding vascular receptors to enhance the contractility of resistance arterioles and increase blood pressure (18, 19). In addition, elevated stress hormone levels trigger the inflammatory and oxidative stress pathways by activating the nicotinamide adenine dinucleotide phosphate oxidase uncoupling of endothelial/neuronal nitric oxide synthase, thereby inducing endothelial and neuronal dysfunction (20).

Some epidemiological studies have suggested associations between traffic noise and hypertension, with one study relating aircraft noise and road traffic noise to an increased risk of hypertension (10, 11). However, epidemiological investigations conducted in the working population have not yet reached a consistent conclusion (12–15). Differences in findings have been attributed to differences in study design, sample sizes and baseline characteristics, and the influence of confounding factors. Meta-analyses have been used to summarize the relationship between occupational noise exposure and the risk of hypertension. However, their conclusions have been limited. Meta-analyses conducted by Wang et al. (21) and Yang et al. (22) included only Chinese literature and Chinese workers. Different study designs (cross-sectional studies, cohort studies, and case-control studies) were included in the three meta-analyses (23–25), which may indirectly explain the results. Recent meta-analyses have been limited by the overall low methodological quality of the included epidemiological studies, with only three of the 24 studies having a low risk of bias (26).

The purpose of this study was to explore the relationship between occupational noise exposure and hypertension risk, to provide a theoretical basis for improving conditions for factory workers and promoting workplace protection.

## Methods

This was a case-control study. It was conducted according to the Strengthening the Reporting of Observational Studies in Epidemiology (STROBE) reporting guidelines (27) (Supplementary Table S1).

### Study Design and Population

Male workers were recruited from a modern automobile manufacturing facility in Shenyang. Participants underwent occupational health examinations between July and October 2013. The inclusion criteria of participants were as follows: (1) Those working in places where occupational noise may exist; (2) Those with a cumulative duration of noise exposure of ≥1 year; (3) Those who were physically healthy. Participants were excluded if they were new employees, worked in an office space free of occupational noise (e.g., finance department, management department, etc.), or those who suffered from coronary heart disease, liver disease, kidney disease, or any endocrine disease. Hypertension was defined as a systolic blood pressure of ≥140 mmHg and or a diastolic blood pressure of ≥90 mmHg (2018 ESC/ESH Practice Guidelines) (28). Participants who met the criteria for hypertension were assigned to the case group, otherwise, they were placed in the control group.

This study was approved by the medical scientific research ethics committee of the First Affiliated Hospital of China Medical University (No. AF-SOP-07-1.0-01). All procedures performed in the study complied with the ethical standards of the institution, the National Research Committee, and the 1964 Helsinki Declaration and its subsequent amendments, or similar ethical standards.

### Data Collection

The patients and the public were not involved in the design and analysis of this study. Demographic and clinical examination data were extracted from the management system of the medical examination center. Occupational noise exposure was measured in the workplace where the members of factory personnel were located, and measurements were undertaken by an occupational health assessment institution with the relevant safety assessment qualification.

Basic participant information was collected by two trained interviewers using a questionnaire survey that included their age, family history of hypertension (yes/no), smoking status (yes/no), job role, and duration in post. Smoking was defined as at least 1 cigarette per day for a year or more.

Clinical examination and blood sample collection were conducted by a healthcare professional. The clinical examination included height (m), weight (kg), body mass index (BMI), blood pressure (mmHg), listening test (normal/abnormal), and pulmonary function (normal/abnormal). Normal pulmonary function was defined using the ratio of forced expiratory volume in 1 s (FEV1) to forced vital capacity (FVC) taking a value of >80 %. Height and weight were measured using an automatic measuring instrument, and body mass index (BMI) was calculated as weight/height^2^ (kg/m^2^). Blood pressure was measured in the upper left arm after 5–10 m of rest in a seated position using an automated device (HEM-746C, Omron, Japan). Each of the above was measured twice, and the mean of the two measurements was used in the analysis. Blood tests were conducted using an automatic biochemical instrument operated by trained technicians, including total cholesterol (TC), triglyceride (TG), high-density lipoprotein (HDL), and low-density lipoprotein (LDL) levels.

Occupational noise exposure was measured by occupational safety inspection professionals, using a statistical noise analyzer (AWA6218; Beijing Xihua Instrument Technology Co., Ltd.). Actual noise levels were recorded according to the normalization of the equivalent continuous A-weighted sound pressure level to a nominal 8-hour working day (LEX, 8h).

### Statistical Analysis

In the presence of any missing data, multiple imputation was used to complete the dataset. Continuous variables were shown as median and inter-quartile ranges (25, 75%), and categorical variables were shown as frequencies (%). Mann-Whitney U-tests and chi-square tests, respectively, were used to compare the distributions of continuous and categorical variables between the two groups.

The odds ratio (OR) and 95% confidence interval (CI) of each variable were calculated using logistic regression. A univariate model was used to describe the linear effects of continuous variables. The continuous variables that were included in the corresponding risk models were transformed into categorical variables by selecting the corresponding critical values to ensure the clinically relevant balance between the groups (29). For occupational noise, the critical value was <85 dB(A), and ≥85 dB(A). For age, thresholds of <24, 24–25, 26–29 and ≥30 years were used. For BMI, the critical value was <18.55, 18.55-23.98, and ≥23.99 kg/m^2^. For heart rate, the critical value was <60, 60–100, and ≥100 beats per minute. For TG, the critical value was <1.70, 1.70–2.25, and ≥2.26 mmol/L. For TC, the critical value was <5.18, 5.18-6.21, and ≥6.22 mmol/L. For LDL, the critical value was <3.37, 3.37–4.13, and ≥4.14 mmol/L. For HDL, the critical value was <1.04, 1.04–1.54, and ≥1.55 mmol/L (30). Years of working at the facility were included as a continuous variable. Smoking status (reference: none), family history (reference: none), pulmonary function (reference: normal), and hearing (reference: normal) were also included in the risk model.

To explore the accuracy of using occupational noise as a predictor of hypertension, the association was explored according to the inter-quartile range of occupational noise levels [ ≤ 76.4 dB(A), 76.41–78.8 dB(A), 78.81–80.90 dB(A), and >80.90 dB(A)]. Finally, Restricted cubic splines (RCS) with knots at the 25, 50, and 75th percentiles of the distribution were used to assess the dose-effect relationship between them, with 80 dB(A) as the reference group. A Spearman correlation test was performed to evaluate the association between occupational noise level and hypertension.

As participants were not randomly assigned to case and control groups, therefore, we used propensity score matching (PSM) to minimize the impact of confounds on the sensitivity analysis (31, 32). We then calculated the propensity score of each participant using multivariate logistic regression modeling. A nearest-neighbor matching method was then used, making the ratio between the case group and control group 1:2, that is, each 1 participant from the case group was matched to 2 participants in the control group with similar propensity scores. The balance of the matched model was assessed using the standardized mean differences between the two groups (33). Finally, the corresponding OR and 95% CI of noise exposure on the risk of hypertension were calculated using univariate logistic regression.

Statistical analyses were conducted using SPSS version 25 (Armonk, NY: IBM Corp.) (34), except for spline fitting, which was performed using SAS version 9.4 (SAS Institute, Cary, NC). A two-sided *P*-value < 0.05 was considered statistically significant.

## Result

### Demographic Characteristics

Table 1 shows the participant characteristics of the case and control groups across the entire study cohort. There were 500 cases and 4,356 controls. Noise exposure level, age, TG, TC, LDL, BMI, heart rate, years working at the facility, and pulmonary function were higher in the case group than in the control group (*P* < 0.05). HDL was lower in the case group than in the control group (*P* < 0.05). Smoking, family history of hypertension, and hearing level did not significantly differ between the two groups (*P* > 0.05).

**Table 1:** Baseline characteristics of the groups in all participants.

	**Total = 4,856**	**Case (*n* = 500)**	**Control (*n* = 4,356)**	***P*-value**
Noise [dB(A)]	78.8(76.4–80.9)	78.8(78.8–81.1)	78.8(75.9–80.6)	<0.001^*^
**Demographics**				
Age (year)	26(24–30)	28.5(25.5–32)	26(24–29)	<0.001^*^
Years of working (year)	2(1–3)	2(1–6)	2(1–3)	<0.001^*^
Smoking				0.57
No	2,020(41.6%)	202(40.4%)	1,818(41.7%)	
Yes	2,836(58.4%)	298(59.6%)	2,538(58.3%)	
Family history of hypertension				0.90
No	84(1.7%)	9(1.8%)	75(1.7%)	
Yes	4,772(98.3%)	491(98.2%)	4,281(98.3%)	
Clinical examination				
Heart rate (beats per minute)	79(72–87)	83.5(77–94)	78.5(72–86)	<0.001^*^
BMI (kg/m^2^)	23.95(21.3–26.81)	27.68(24.79–29.48)	23.66(21.11–26.23)	<0.001^*^
Hearing				0.89
Normal	4,629(95.3%)	476(95.2%)	4,153(95.3%)	
Abnormal	227(4.7%)	24(4.8%)	203(4.7%)	
Pulmonary function				0.001^*^
Normal	4,651(95.8%)	465(93.0%)	4,186(96.1%)	
Abnormal	205(4.2%)	35(7.0%)	170(3.9%)	
**Blood biochemistry**				
TG (mmol/L)	1.27(0.84–1.9)	1.76(1.22–2.73)	1.2(0.81–1.83)	<0.001^*^
TC (mmol/L)	4.4(3.9–5)	4.9(4.2–5.5)	4.4(3.8–5.0)	<0.001^*^
LDL (mmol/L)	2.51(2.08–2.94)	2.78(2.35–3.24)	2.47(2.06–2.91)	<0.001^*^
HDL (mmol/L)	1.07(0.94–1.21)	1.02(0.91–1.15)	1.07(0.95–1.22)	<0.001^*^

*TG, triglyceride; HDL, high-density lipoprotein; LDL, low-density lipoprotein; TC, total cholesterol; BMI, body mass index. The differences in the distribution of noise, age, heart rate, TG, HDL, LDL, TC, BMI, and years of working between the two groups by Mann-Whitney U tests. The differences in the distribution of smoke, family history of hypertension, hearing, pulmonary function between the two groups by Chi square test*.

* *P < 0.05*.

### Identify Predictors of Hypertension

Independent predictors of hypertension in the entire participant cohort were identified using logistic regression models with the original value for each variable (Supplementary Table S2). In the multivariate logistic regression, an increase in occupational noise exposure level of 1 dB(A) was associated with an increased hypertension risk of 8.3% (OR: 1.083, 95% CI: 1.058–1.109). Age, heart rate, and BMI were independent predictors of the risk of hypertension.

Table 2 shows the independent predictors of hypertension in the entire cohort identified by analyzing the nonlinear effects of the continuous variables. Multivariate logistic regression showed that exposure to occupational noise ≥85 dB(A) was significantly associated with the risk of hypertension (OR: 4.917, 95%CI: 2.920–8.279). When occupational noise level was analyzed as a categorical variable, the risk of having hypertension in the fourth quartile was 1.742 (95% CI: 1.313–2.310), compared to the first quartile. In addition, age, heart rate, BMI, TG, and TC were independent predictors of hypertension.

**Table 2:** The OR and 95% CI of the relationship between classified variables and hypertension in all participants.

	**Univariate model**		**Multivariate model** ^**a**^	
	**OR (95%CI)**	**P value**	**OR (95%CI)**	**P value**
**Noise [dB(A)]**				
Reference: <85	3.953 (2.491–6.274)	<0.001^*^	4.917 (2.920–8.279)	<0.001^*^
**Noise [dB(A)]**				
Reference: ≤ 76.40	1	0.004^*^	1	<0.001^*^
76.41–78.80	1.096 (0.850–1.413)	0.48	0.955 (0.730–1.250)	0.739
78.81–80.90	1.208 (0.894–1.632)	0.22	1.073 (0.781–1.475)	0.66
>80.90	1.570 (1.206–2.046)	0.001^*^	1.742 (1.313–2.310)	<0.001^*^
**Demographics**				
**Age(year)**				
Reference: <24	1	<0.001^*^	1	<0.001^*^
24–25	1.440 (1.041–1.994)	0.03^*^	1.402 (0.998–1.969)	0.05
26–29	1.421 (1.038–1.945)	0.03^*^	1.098 (0.787–1.530)	0.58
≥30	3.292 (2.468–4.390)	<0.001^*^	2.094 (1.503–2.917)	<0.001^*^
Years of working (year)	1.068 (1.039–1.098)	<0.001^*^	1.001 (0.968–1.035)	0.954
**Smoking**				
Reference: No	1.057 (0.875–1.276)	0.57	1.038 (0.847–1.272)	0.72
**Family history of hypertension**				
Reference: No	0.956 (0.476–1.920)	0.9	0.802 (0.376–1.714)	0.57
**Clinical examination**				
**Heart rate (beats per minute)**				
Reference: 60–99	1	<0.001^*^	1	<0.001^*^
<60	0.492 (0.179–1.351)	0.17	0.631 (0.223–1.786)	0.39
≥100	3.679 (2.739–4.941)	<0.001^*^	4.018 (2.889–5.586)	<0.001^*^
BMI (kg/m^2^)
Reference: 18.55– 23.89	1	<0.001^*^	1	<0.001^*^
<18.55	0.406 (0.148–1.116)	0.08	0.443 (0.159–1.234)	0.12
≥23.99	4.879 (3.855–6.221)	<0.001^*^	3.942 (3.047–5.100)	<0.001^*^
**Hearing**				
Reference: Normal	1.032 (0.669–1.591)	0.89	0.897 (0.564–1.427)	0.65
**Pulmonary function**				
Reference: Normal	1.853 (1.272–2.700)	0.001^*^	1.457 (0.964–2.204)	0.07
**Blood biochemistry**				
**TG (mmol/L)**				
Reference: <1.70	1	<0.001^*^	1	<0.001^*^
1.70–2.25	1.896 (1.462–2.460)	<0.001^*^	1.180 (0.888–1.569)	0.25
≥2.26	3.456 (2.796–4.272)	<0.001^*^	1.699 (1.302–2.216)	<0.001^*^
**TC (mmol/L)**				
Reference: <5.18	1	<0.001^*^	1	0.02^*^
5.18–6.21	2.321 (1.872–2.877)	<0.001^*^	1.365 (1.027–1.815)	0.03^*^
≥6.22	4.391 (3.159–6.103)	<0.001^*^	1.923 (1.165–3.174)	0.01^*^
**LDL (mmol/L)**				
Reference: <3.37	1	<0.001^*^	1	0.39
3.37–4.13	2.288 (1.773–2.952)	<0.001^*^	1.117 (0.789–1.582)	0.53
≥4.14	3.800 (2.301–6.275)	<0.001^*^	1.606 (0.812–3.175)	0.17
**HDL (mmol/L)**				
Reference: <1.04	1	<0.001^*^	1	0.84
1.04–1.54	2.139 (1.076–4.252)	0.03^*^	0.938 (0.450–1.954)	0.86
≥1.55	1.486 (0.747–2.957)	0.26	1.001 (0.486–2.062)	0.99

*OR, odds ratio; CI, confidence interval; TG, triglyceride; HDL, high-density lipoprotein; LDL, low-density lipoprotein; TC, total cholesterol; BMI, body mass index*.

a *Multivariable models include noise, age, years of working, smoking, family history, heart rate, BMI, hearing, pulmonary function, TG, TC, LDL, HDL*.

* *P < 0.05*.

### Sensitivity Analysis

PSM was used for sensitivity analysis to minimize the effects of confounding factors. The baseline characteristics of participants after PSM are shown in Table 3. When occupational noise was analyzed as either a continuous or categorical variable, it was significantly associated with the risk of hypertension, consistent with the results of the other analyses performed (Table 4).

**Table 3:** Baseline characteristics of the groups in PSM participants.

	**Total = 1,500**	**Case (*n* = 500)**	**Control (*n* = 1,000)**	**Standardized mean differences**		**P–value**
				**Before PSM**	**After PSM**	
Noise [dB(A)]	78.8(78.2–80.9)	78.8(78.8–81.1)	78.8(75.9–80.6)	/	/	<0.001^*^
Demographics				/	/	
Age (year)^**b**^		28.5(25–32)	28(25–32)	0.43	0.04	0.96
Years of working (year)	2(1–7)	2(1–6)	2(1–7)	/	/	0.24
Smoking				/	/	0.97
No	607(40.5%)	202(40.4%)	405(40.5%)	/	/	
Yes	893(59.5%)	298(59.6%)	595(59.5%)	/	/	
Family history of hypertension				/	/	0.66
No	24(1.6%)	9(1.8%)	15(1.5%)	/	/	
Yes	1,476(98.4%)	491(98.2%)	985(98.5%)	/	/	
Clinical examination				/	/	
Heart rate (beats per minute)^**b**^	83(77–92)	83.5(77–94)	83(77–92)	0.48	0.06	0.41
BMI (kg/m^2^)^**b**^	27.1(24.68–29.48)	27.68(24.79–29.48)	27.02(24.5–29.48)	0.93	0.05	0.26
Hearing				/	/	0.19
Normal	1,411(94.1%)	476(95.2%)	935(93.5%)	/	/	
Abnormal	89(5.9%)	24(4.8%)	65(6.5%)	/	/	
Pulmonary function				/	/	0.11
Normal	1,415(94.3%)	465(93%)	950(95%)	/	/	
Abnormal	85(5.7%)	35(7%)	50(5%)	/	/	
Blood biochemistry				/	/	
TG (mmol/L)^**b**^	1.73(1.2–2.6)	1.76(1.22–2.73)	1.71(1.18–2.51)	0.34	0.05	0.23
TC (mmol/L)^**b**^	4.9(4.3–5.5)	4.9(4.2–5.5)	4.85(4.3–5.5)	0.5	0.04	0.64
LDL (mmol/L)	2.77(2.35–3.24)	2.78(2.35–3.24)	2.77(2.35–3.23)	/	/	0.80
HDL (mmol/L)	1.02(0.9–1.15)	1.02(0.91–1.15)	1.01(0.9–1.15)	/	/	0.56

*PSM, propensity score matching; TG, triglyceride; HDL, high-density lipoprotein; LDL, low-density lipoprotein; TC, total cholesterol; BMI, body mass index. The differences in the distribution of noise, age, heart rate, TG, HDL, LDL, TC, BMI, and years of working between the two groups by Mann–Whitney U tests. The differences in the distribution of smoke, family history of hypertension, hearing, pulmonary function between the two groups by Chi square test*.

b *Those variables (age, heart rate, BMI, TG, TC) that has been identified as independent predictors of hypertension*.

* *P < 0.05*.

**Table 4:** The OR and 95% CI of the relationship between noise and hypertension in PSM participants.

	**Univariate model**	
	**OR (95%CI)**	***P* value**
Noise, per 1 dB(A)	1.068(1.040–1.096)	<0.001^*^
**Noise:**		
Reference: <85	3.114(1.698–5.711)	<0.001^*^
**Noise(dB)**		
Reference: ≤ 76.40	1	0.001^*^
76.41–78.80	1.138(0.851–1.521)	0.384
78.81–80.90	1.054(0.749–1.483)	0.762
>80.90	1.797(1.316–2.454)	<0.001^*^

*OR, odds ratio; CI, confidence interval*.

* *P < 0.05*.

### Dose Effect Relationship Between Noise and Hypertension

**Figure 1:**
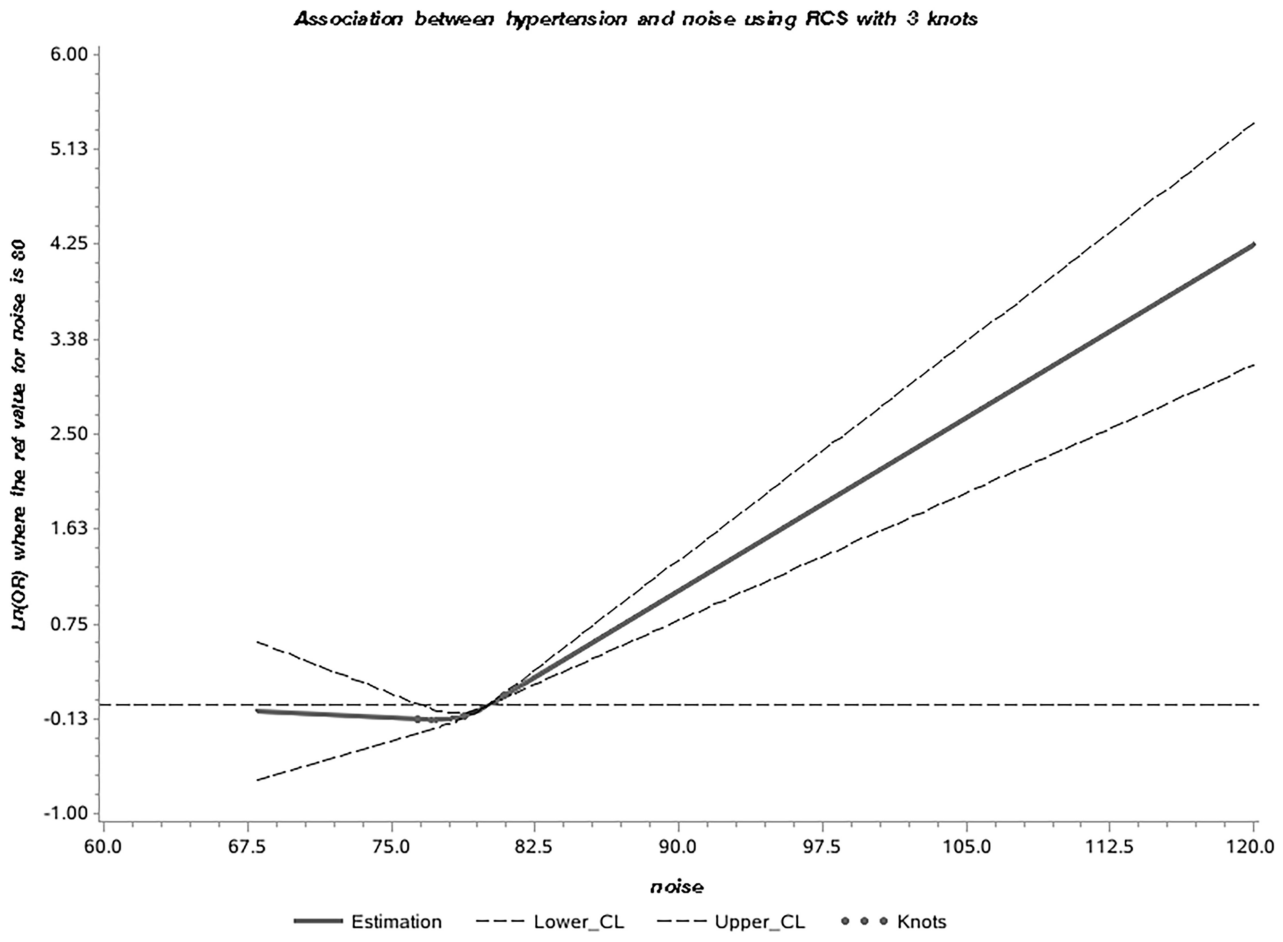
Dose effect relationship between occupational noise and hypertension in entire participants.

The Spearman correlation test showed that the correlation between occupational noise exposure and hypertension was significant at the 0.01 level (2-tailed). Subsequently, the RCS curve showed a positively correlated nonlinear dose-response relationship between noise exposure and hypertension (*P*_nonlinear_ = 0.002; Figure 1). The risk of hypertension was relatively stable until a predicted occupational noise level of around 80 dB(A) and then started to increase rapidly afterward.

## Discussion

### Main Results and Description

We used a case-control study design to explore predictors of hypertension. Independent of whether occupational noise was analyzed as a continuous or categorical variable, there was a nonlinear dose-response relationship between it and hypertension.

### Literature Review

We found that occupational noise exposure was associated with an increased risk of hypertension. Previous studies have reported that occupational noise exposure increases the risk of hypertension. A cross-sectional study reported this in steelworkers (OR: 2.03, 95% CI: 1.15–3.58) (12), Bolm et al. (26) conducted a meta-analysis of 24 studies and concluded that the risk of hypertension increased 1.77-fold (95% CI 1.36–2.29) for employees exposed to occupational noise levels of 80–85 dB(A), and increased 3.50-fold (95% CI 1.56–7.86) for employees exposed at 85–90 dB(A). However, Tessier et al. (13) and Stokholm et al. (14) reported an absence of any such association. The inconsistency of these findings may be attributed to the noise exposures reported in their studies not being high enough to detect an effect. In addition, the present results show a nonlinear dose-response relationship between occupational noise and the risk of hypertension. For occupational noise exposures in the range 68–80 dB(A), the risk of hypertension was relatively stable. Exposures above 80 dB(A) were associated with a risk of hypertension that increased rapidly with noise exposure level. Lin et al. (15) also reported a curved exposure-response pattern between occupational noise and hypertension, whereby the risk of hypertension increased for exposures between 82 and 106 dB(A), and then sharply decreased for exposures over 107 dB(A). This may be related to the extensive use of personal protective equipment in people exposed to high levels of occupational noise, thereby reducing the incidence of hypertension.

### Study Strengths and Limitations

Hypertension is a consequence of many factors working together. The influence of potential confounding factors on research findings must be considered when analyzing the impact of noise exposure on the risk of hypertension. Multifactor regression analysis is the most commonly used method that adjusts for confounding factors, but collinearity between variables can lead to instabilities in such a model (35). Firstly, we used PSM to balance the distributions of potentially confounding variables between participant groups by matching participants in the case group with those from the control group, thereby reducing the error (36). Secondly, we used RCR regression to flexibly model the association between occupational noise and the risk of hypertension. As such, we obtained a more intuitive dose-effect relationship curve.

Our study also had some limitations. Firstly, we have no information about the use of antihypertensive drugs within the participant cohort, which may provide a confounding factor to determining the association between occupational noise exposure and hypertension. Secondly, we did not set the caliper value for the PSM, potentially leading to a deviation in the results (37). However, the balance achieved between the groups after PSM suggests that the model was stable. In addition, noise exposure and blood pressure measurements were collected at a single time point, so it is not possible to infer any causal relationship between noise exposure and hypertension. Prospective longitudinal studies will be needed to explore the causal relationship between noise exposure and hypertension.

## Data Availability Statement

The raw data supporting the conclusions of this article will be made available by the authors, without undue reservation.

## Ethics Statement

This study has been approved by the medical scientific research Ethics Committee of the First Affiliated Hospital of China Medical University (No. AF-SOP-07-1.0-01). Written informed consent for participation was not required for this study in accordance with the national legislation and the institutional requirements.

## Author Contributions

XW, CL, and BZ contributed to conception and design of the study. XZ and YS organized the database. BZ and XW performed the statistical analysis. CL wrote the first draft of the manuscript. XW, CL, YS, and XZ wrote sections of the manuscript. XW, CL, DZ, and YL revised various parts of the manuscript. All authors contributed to manuscript revision, read, and approved the submitted version.

## Conflict of Interest

The authors declare that the research was conducted in the absence of any commercial or financial relationships that could be construed as a potential conflict of interest.

## Publisher's Note

All claims expressed in this article are solely those of the authors and do not necessarily represent those of their affiliated organizations, or those of the publisher, the editors and the reviewers. Any product that may be evaluated in this article, or claim that may be made by its manufacturer, is not guaranteed or endorsed by the publisher.
