# Supplementary material for: The Impact of Occupational Noise on Hypertension Risk: A Case-Control Study in Automobile Factory Personnel
Source: Front Cardiovasc Med. 2022 Feb 17;9:803695. doi: 10.3389/fcvm.2022.803695 (PMC8891519; doi:10.3389/fcvm.2022.803695)
Supplement: Supplementary file 1 [file Table_1.DOCX]

Supplementary Table

Supplementary Table S1 STROBE Statement—Checklist of items that should be included in reports of case-control studies.

Supplementary Table S2 The OR and 95% CI of the relationship between the original value of each variable and hypertension in all participants.

Supplementary Table 1 STROBE Statement—Checklist of items that should be included in reports of case-control studies.

|  | Item No | Recommendation | Page No |
| --- | --- | --- | --- |
| **Title and abstract** | 1 | (*a*) Indicate the study’s design with a commonly used term in the title or the abstract | 1 |
|  |  | (*b*) Provide in the abstract an informative and balanced summary of what was done and what was found | 1-2 |
| Introduction | | | |
| Background/rationale | 2 | Explain the scientific background and rationale for the investigation being reported | 2-3 |
| Objectives | 3 | State specific objectives, including any prespecified hypotheses | 3 |
| Methods | | | |
| Study design | 4 | Present key elements of study design early in the paper | 3 |
| Setting | 5 | Describe the setting, locations, and relevant dates, including periods of recruitment, exposure, follow-up, and data collection | 3-4 |
| Participants | 6 | (*a*) Give the eligibility criteria, and the sources and methods of case ascertainment and control selection. Give the rationale for the choice of cases and controls | 3 |
|  |  | (*b*) For matched studies, give matching criteria and the number of controls per case | / |
| Variables | 7 | Clearly define all outcomes, exposures, predictors, potential confounders, and effect modifiers. Give diagnostic criteria, if applicable | 4 |
| Data sources/ measurement | 8* | For each variable of interest, give sources of data and details of methods of assessment (measurement). Describe comparability of assessment methods if there is more than one group | 4-5 |
| Bias | 9 | Describe any efforts to address potential sources of bias | 5 |
| Study size | 10 | Explain how the study size was arrived at | 3-4 |
| Quantitative variables | 11 | Explain how quantitative variables were handled in the analyses. If applicable, describe which groupings were chosen and why | 5 |
| Statistical methods | 12 | (*a*) Describe all statistical methods, including those used to control for confounding | 5 |
|  |  | (*b*) Describe any methods used to examine subgroups and interactions | 5 |
|  |  | (*c*) Explain how missing data were addressed | 5 |
|  |  | (*d*) If applicable, explain how matching of cases and controls was addressed | 5 |
|  |  | (*e*) Describe any sensitivity analyses | 5 |
| Results | | | |
| Participants | 13* | (a) Report numbers of individuals at each stage of study—eg numbers potentially eligible, examined for eligibility, confirmed eligible, included in the study, completing follow-up, and analysed | 6 |
|  |  | (b) Give reasons for non-participation at each stage | 3 |
|  |  | (c) Consider use of a flow diagram | / |
| Descriptive data | 14* | (a) Give characteristics of study participants (eg demographic, clinical, social) and information on exposures and potential confounders | Table 1  Table 3 |
|  |  | (b) Indicate number of participants with missing data for each variable of interest | / |
| Outcome data | 15* | Report numbers in each exposure category, or summary measures of exposure | Table 2 |
| Main results | 16 | (*a*) Give unadjusted estimates and, if applicable, confounder-adjusted estimates and their precision (eg, 95% confidence interval). Make clear which confounders were adjusted for and why they were included | Table 2  Table 4 |
|  |  | (*b*) Report category boundaries when continuous variables were categorized | Table 2 |
|  |  | (*c*) If relevant, consider translating estimates of relative risk into absolute risk for a meaningful time period | Table 2 |
| Other analyses | 17 | Report other analyses done—eg analyses of subgroups and interactions, and sensitivity analyses | 7  Table 3  Table 4 |
| Discussion | | | |
| Key results | 18 | Summarise key results with reference to study objectives | 7 |
| Limitations | 19 | Discuss limitations of the study, taking into account sources of potential bias or imprecision. Discuss both direction and magnitude of any potential bias | 8 |
| Interpretation | 20 | Give a cautious overall interpretation of results considering objectives, limitations, multiplicity of analyses, results from similar studies, and other relevant evidence | 8 |
| Generalisability | 21 | Discuss the generalisability (external validity) of the study results | 8 |
| Other information | | | |
| Funding | 22 | Give the source of funding and the role of the funders for the present study and, if applicable, for the original study on which the present article is based | NA |

Supplement Table S2 The OR and 95% CI of the relationship between the original value of each variable and hypertension in all participants.

|  | Univariate model |  | Multivariate model **^a^** |  |
| --- | --- | --- | --- | --- |
|  | OR (95%CI) | P value | OR (95%CI) | P value |
| Noise, per 1 dB(A) | 1.077(1.055-1.100) | ＜0.001* | 1.083(1.058-1.109) | ＜0.001* |
| Demographics |  |  |  |  |
| Age(year) | 1.092(1.074-1.111) | ＜0.001* | 1.080(1.057-1.103) | ＜0.001* |
| Years of working (year) | 1.068(1.039-1.098) | ＜0.001* | 0.992(0.959-1.026) | 0.62 |
| Smoking |  |  |  |  |
| Reference: No | 1.057(0.875-1.276) | 0.57 | 0.989(0.802-1.217) | 0.91 |
| Family history of hypertension |  |  |  |  |
| Reference: No | 0.956(0.476-1.920) | 0.9 | 0.740(0.346-1.583) | 0.44 |
| Clinical examination |  |  |  |  |
| Heart rate (beats per minute) | 1.044(1.036-1.052) | ＜0.001* | 1.044(1.035-1.053) | ＜0.001* |
| BMI (kg/m2) | 1.252(1.221-1.283) | ＜0.001* | 1.225(1.191-1.260) | ＜0.001* |
| Hearing |  |  |  |  |
| Reference: Normal | 1.032(0.669-1.591) | 0.89 | 0.762(0.470-1.236) | 0.27 |
| Pulmonary function |  |  |  |  |
| Reference: Normal | 1.853(1.272-2.700) | 0.001* | 1.513(0.988-2.317) | 0.06 |
| Blood biochemistry |  |  |  |  |
| TG (mmol/L) | 1.246(1.185-1.310) | ＜0.001* | 1.071(0.976-1.176) | 0.15 |
| TC (mmol/L) | 1.702(1.551-1.868) | ＜0.001* | 1.072(0.780-1.472) | 0.67 |
| LDL (mmol/L) | 1.922(1.84-2.193) | ＜0.001* | 1.251(0.846-1.851) | 0.27 |
| HDL (mmol/L) | 0.295(0.184-0.472) | ＜0.001* | 1.228(0.639-2.357) | 0.54 |

OR: odds ratio, CI: confidence interval, TG: triglyceride, HDL: high-density lipoprotein, LDL: low-density lipoprotein, TC: total cholesterol, BMI: body mass index.

**a**: Multivariable models include noise, age, years of working, smoking, family history, heart rate, BMI, hearing, pulmonary function, TG, TC, LDL, HDL.

* P＜0.05
